# Most Caenorhabditis elegans microRNAs Are Individually Not Essential for Development or Viability

**DOI:** 10.1371/journal.pgen.0030215

**Published:** 2007-12-14

**Authors:** Eric A Miska, Ezequiel Alvarez-Saavedra, Allison L Abbott, Nelson C Lau, Andrew B Hellman, Shannon M McGonagle, David P Bartel, Victor R Ambros, H. Robert Horvitz

**Affiliations:** 1 Department of Biology, Massachusetts Institute of Technology, Cambridge, Massachusetts, United States of America; 2 Howard Hughes Medical Institute, Massachusetts Institute of Technology, Cambridge, Massachusetts, United States of America; 3 Department of Genetics, Dartmouth Medical School, Hanover, New Hampshire, United States of America; 4 Whitehead Institute for Biomedical Research, Cambridge, Massachusetts, United States of America; University of California San Francisco Diabetes Center, United States of America

## Abstract

MicroRNAs (miRNAs), a large class of short noncoding RNAs found in many plants and animals, often act to post-transcriptionally inhibit gene expression. We report the generation of deletion mutations in 87 miRNA genes in Caenorhabditis elegans, expanding the number of mutated miRNA genes to 95, or 83% of known C. elegans miRNAs. We find that the majority of miRNAs are not essential for the viability or development of C. elegans, and mutations in most miRNA genes do not result in grossly abnormal phenotypes. These observations are consistent with the hypothesis that there is significant functional redundancy among miRNAs or among gene pathways regulated by miRNAs. This study represents the first comprehensive genetic analysis of miRNA function in any organism and provides a unique, permanent resource for the systematic study of miRNAs.

## Author Summary

MicroRNAs (miRNAs) are tiny endogenous RNAs that regulate gene expression in plants and animals. Individual miRNAs have important roles in development, immunity, and cancer. Although the investigation of miRNA function is of great importance, to date few miRNAs have been studied in the intact organism because of a lack of mutants in which specific miRNAs have been inactivated. Here we describe a collection of loss-of-function mutants representing the majority of all known miRNA genes in the nematode Caenorhabditis elegans. This study identifies a new role for miRNAs in C. elegans and also demonstrates that most miRNAs are not essential for viability or development. Our findings suggest that many miRNAs act redundantly with other miRNAs or other pathways. We expect that this collection of miRNA mutants will become a widely used resource to further our understanding of the biology of miRNAs.

## Introduction

MicroRNAs (miRNAs) were discovered in C. elegans during studies of the control of developmental timing [1–5]. miRNAs are approximately 22-nucleotide noncoding RNAs that are thought to regulate gene expression through sequence-specific base-pairing with target mRNAs [6]. miRNAs have been identified in organisms as diverse as roundworms, flies, fish, frogs, mammals, flowering plants, mosses, and even viruses, using genetics, molecular cloning, and predictions from bioinformatics [7–16]. In C. elegans about 115 miRNA genes have been confidently identified [10,11,17–20].

In animals, miRNAs are transcribed as long RNA precursors (pri-miRNAs), which are processed in the nucleus by the RNase III enzyme complex Drosha-Pasha/DGCR8 to form the approximately 70-base pre-miRNAs [21–25] or are derived directly from introns [26,27]. Pre-miRNAs are exported from the nucleus by Exportin-5 [28], processed by the RNase III enzyme Dicer, and incorporated into an Argonaute-containing RNA-induced silencing complex (RISC) [29]. Within the silencing complex, metazoan miRNAs pair to the mRNAs of protein-coding genes, usually through imperfect base-pairing with the 3′-UTR, thereby specifying the posttranscriptional repression of these target mRNAs [6,30]. Binding of the silencing complex causes translational repression [31–33] and/or mRNA destabilization, which is sometimes through direct mRNA cleavage [34,35], but usually through other mechanisms [36–40]. Because many messages have been under selective pressure to preserve pairing to a 6mer in the 5′ region of the miRNA known as the miRNA seed (nucleotides 2–7), targets of metazoan miRNAs can be predicted above the background of false-positives by searching for conserved matches to the seed region [41–45]. In nematodes, at least 10% of the protein-coding messages appear to be conserved targets of miRNAs [46].

The in vivo functions of a few miRNAs have been established. In C. elegans, the *lin-4* miRNA and the *let-7* family of miRNAs control the timing of aspects of larval development. For example, the *lin-4* miRNA controls hypodermal cell-fate decisions during early larval development by negatively regulating the *lin-14* and *lin-28* mRNAs [1–3,5,47]. The *let-7* miRNA controls hypodermal cell-fate decisions during late-larval development by regulating the *lin-41*, *hbl-1*, *daf-12*, and *pha-4* mRNAs [48–51]. Three additional *C. elegans let-7*-like miRNAs, miR-48, miR-84, and miR-241, also act in the control of developmental timing and likely regulate the *hbl-1* mRNA, but act earlier in development than the *let-7* miRNA [52,53]. The *C. elegans lsy-6* miRNA acts in the asymmetric differentiation of the left and right ASE chemosensory neurons. Specifically, the *lsy-6* miRNA targets the *cog-1* mRNA, resulting in a shift of marker gene expression in the left ASE to resemble marker gene expression in the right ASE [20]. The first miRNA studied functionally in *Drosophila* is encoded by the *bantam* locus, which had previously been identified in a screen for deregulated tissue growth [54]. The *bantam* miRNA stimulates cell proliferation and reduces programmed cell death. *bantam* directly regulates the pro-apoptotic gene *hid*. A second *Drosophila* miRNA, miR-14, also reduces programmed cell death [55]. The muscle-specific *Drosophila* miRNA miR-1 is required for larval development and cardiac differentiation [56,57]. Dmir-7 regulates the transcription factor Yan [58]. Finally, *Drosophila* miR-9a is required for sensory organ precursor specification [59], and *Drosophila* miR-278 is required for energy homeostasis [60]. The first loss-of-function studies of miRNAs in the mouse have been reported demonstrating a role for miR-1 and miR-208 in cardiac growth in response to stress [61,62] and miR-155/BIC in normal immune function [63,64].

miRNA function has also been inferred from studies in which miRNAs have been misexpressed in worms, flies, frogs, mice, and cultured mammalian cells [65]. In addition, miRNA function has been explored by perturbing the functions of genes in the pathway for miRNA biogenesis and by reducing miRNA levels using antisense oligonucleotides. For example, mutants defective in Dicer, which is essential for miRNA biogenesis, have been studied for C. elegans [66,67], *Drosophila* [68,69], the zebrafish [70,71], and the mouse [72–75]. In all cases, Dicer was found to be essential for normal development. In addition, members of the AGO subfamily of Argonaute proteins, which act in the miRNA pathway, are essential for normal C. elegans and mouse development [67,76].

In *Drosophila*, 2′ O-methyl antisense oligoribonucleotides have been used in miRNA depletion studies [77]. This technique was initially described for human cells and C. elegans [78,79] and appears to offer sequence-specific inhibition of small RNAs for a limited time span. Injection of individual 2′ O-methyl antisense oligoribonucleotides complementary to the 46 miRNAs known to be expressed in the fly embryo resulted in a total of 25 different abnormal phenotypes, including defects in patterning, morphogenesis, and cell survival [77]. Knockdown of miRNAs using modified 2′ O-methyl antisense oligoribonucleotides also has been reported for the mouse [80]. Very recently, a study reported the use of morpholinos to knockdown miRNA function in zebrafish and identified a role for miR-375 in pancreatic islet development [81].

To gain a broader understanding of miRNA function*,* we generated a collection of deletion mutants of the majority of known miRNA genes in C. elegans. We found that mutations in most miRNA genes do not result in striking abnormalities, and therefore most miRNA genes likely have subtle or redundant roles. This permanent collection provides a resource for detailed studies of miRNA function not possible previously.

## Results

The cloning of many miRNAs from C. elegans using molecular biological techniques prompted us to take a genetic approach to study miRNA function in vivo in C. elegans through the generation of loss-of-function mutants. We isolated deletion mutants using established C. elegans techniques [82,83]. We made extensive use of the “poison” primer method, which increases the sensitivity of detection of small deletions [84]. Most C. elegans miRNAs were cloned and verified in northern blot experiments [10,11,17,85]. Some miRNAs were predicted based on pre-miRNA folds and verified using northern blotting or PCR with specific primers and cloned miRNA libraries [17,18,85,86]. The public database for miRNAs, miRBase release 9.0, listed 114 C. elegans miRNAs [87,88]. Of these 114, 96 miRNAs are confidently identified, based on expression and the likelihood of being derived from stem-loop precursors, whereas many of the others do not appear to be authentic miRNAs [17–19]. Recently, two studies using high-throughput sequencing methods identified 21 additional miRNAs [19,26] bringing the total number of miRNAs identified with high confidence in C. elegans to 115 and the total number of annotated miRNA candidates to 135.

We isolated knockout mutants covering 87 miRNA genes. We previously described our studies of knockouts of three additional miRNA genes [52], and deletions in two other miRNA genes had been obtained by the C. elegans knockout consortium (D. Moerman, personal communication) [84]. Three miRNA genes had been mutated in genetic screens, *lin-4*, *let-7*, and *lsy-6* [2,4,20]. Thus, 95 C. elegans miRNAs can now be functionally analyzed using mutants (Table 1). Additional alleles for a subset of these miRNA genes were also isolated by the C. elegans knockout consortium (D. Moerman, personal communication) [84].

**Table 1:**
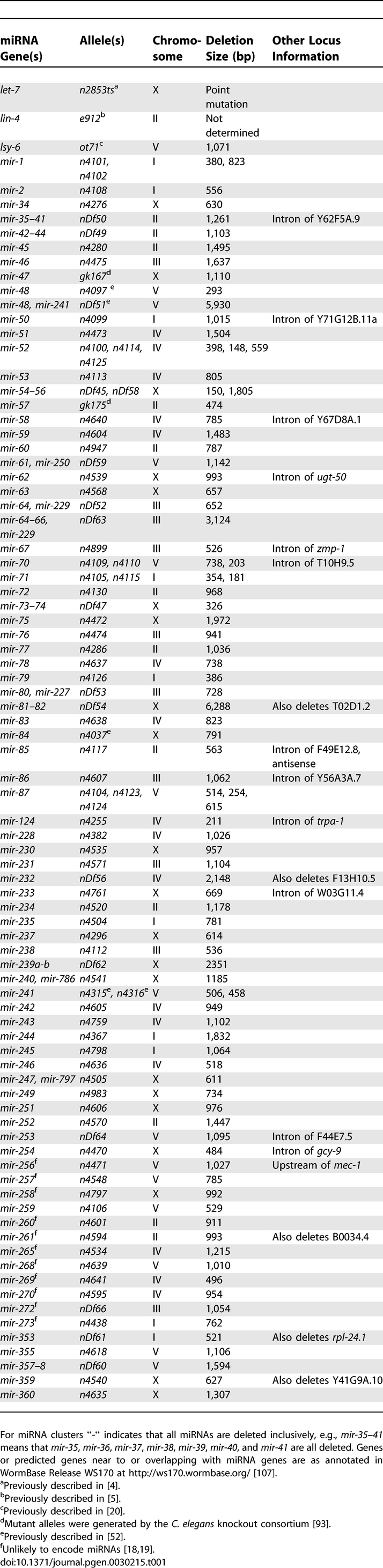
miRNA Mutants

The median size of the deletions we isolated was 911 bases with a range of 181–6,288 bases (Tables 1 and S1). Some deletions likely affect neighboring genes in the case of intergenic miRNA genes or host genes in the case of miRNA genes found in introns. For example, the lethality linked to *mir-50(n4099)* (Table 2) might be a consequence of a loss-of-function of *mir-50* or of an effect on the predicted host gene Y71G12B.11a (Table 1).

**Table 2:**
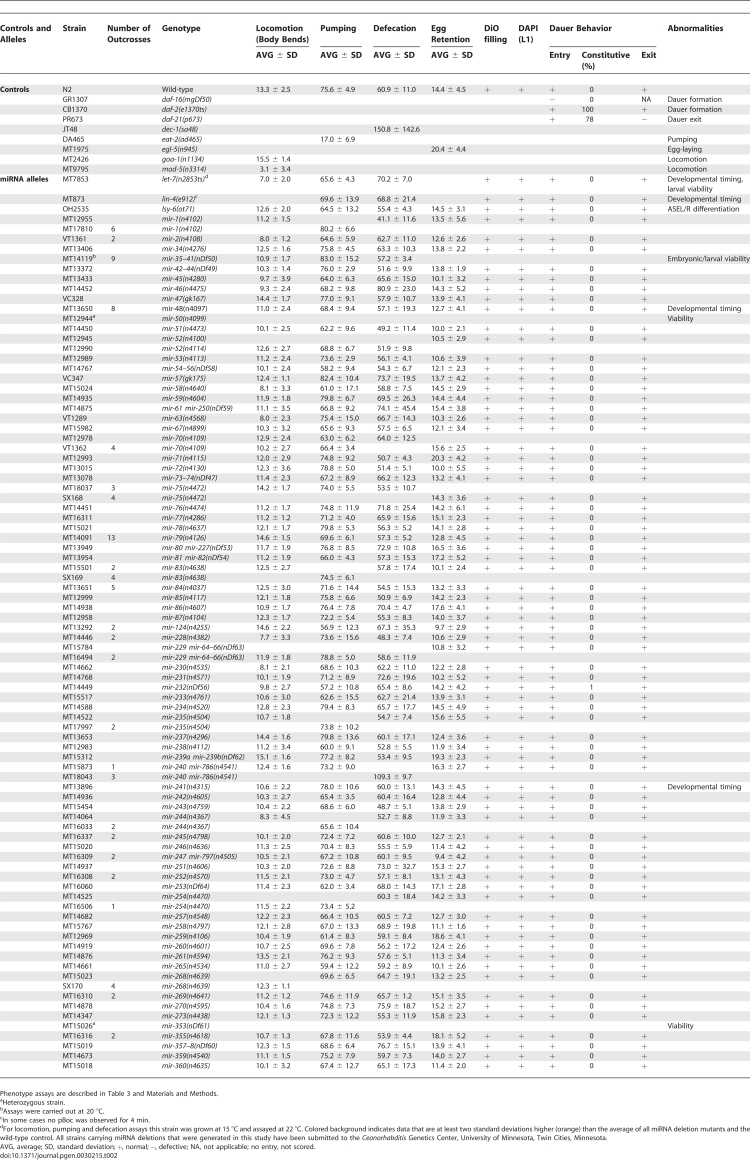
Phenotypic Characterization of miRNA Mutants

**Table 3:**
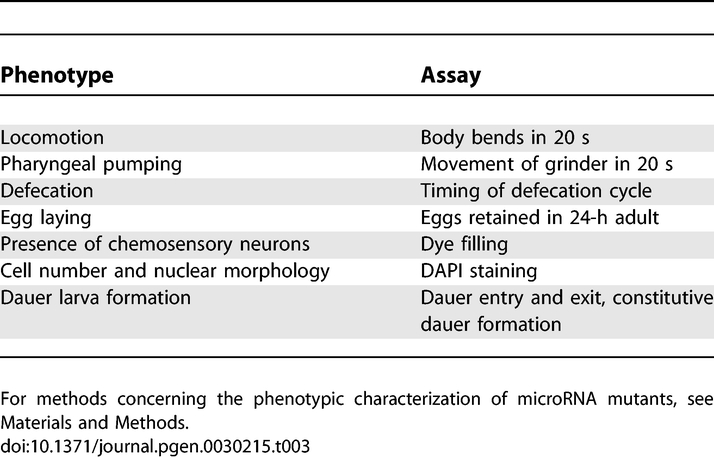
Phenotypes Examined

We performed a broad phenotypic study of all available miRNA loss-of-function mutants, including mutants that had been reported earlier [2,4,20,52]. We focused on phenotypic assays that are relatively rapid and that examine C. elegans morphology, growth, development, and behavior. The assays we performed are shown in Table 3 and the phenotypes we observed are summarized in Table 2. Our initial phenotypic analysis revealed a single new abnormality linked to miRNA loss-of-function: deletion of the *mir-240 mir-797* miRNA cluster resulted in abnormal defecation cycle lengths. This defecation defect was rescued by the introduction of a transgene carrying the *mir-240 mir-797* genomic locus (Table S2). In addition, we observed other abnormal phenotypes. Mutation of the *mir-35–41* miRNA cluster resulted in temperature-sensitive embryonic and larval lethality; this lethality was rescued by the introduction of a transgene carrying the *mir-35–41* genomic locus (unpublished data). We were unable to generate homozygotes for alleles of *mir-50* and *mir-353*. *mir-50* and *mir-353* are in introns of genes that when inactivated by RNAi result in embryonic lethality and may explain why we could not isolate homozygotes for our new deletions. Indeed, the introduction of a transgene carrying the *mir-50* genomic locus failed to rescue the lethality associated with the *mir-50* allele (unpublished data). The number of times each of the deletion strains has been outcrossed is shown in Table 2. It is conceivable that some of the miRNA deletion strains harbor additional mutations that suppress abnormalities conferred by miRNA deletion alleles and that could be revealed by outcrossing. To uncover subtle abnormalities in the miRNA mutant strains will require more detailed analyses, as has been performed for *lin-4*, *let-7*, *lsy-6*, *mir-48*, *mir-84*, and *mir-241*. Nevertheless, we note one striking conclusion: the majority of miRNAs are not essential for C. elegans viability and development.

## Discussion

Here we report the first large-scale collection of miRNA loss-of-function mutants for any organism. We isolated new deletion alleles for 87 miRNA genes. Together with two publicly available deletion mutants, three mutants that we described elsewhere, and three mutants generated in genetic screens, there are now mutants for 95 C. elegans miRNA genes [2,4,20,52]. We hope that this collection will become a widely used resource for the study of miRNA function.

### Loss-of-Function versus Misexpression Studies

The overexpression of the miRNAs miR-84 and miR-61 from transgenes in C. elegans affects vulval development [89,90]. The overexpression of miR-61 leads to the expression in Pn.p cells that do not normally generate vulval cell fates of reporter genes indicative of vulval cell fates [89]. We examined if miR-61 and the closely related miR-247 were required for the normal induction of primary or secondary vulval cell fates by the Pn.p cells. We found that Pn.p cell induction was normal in *mir-61* mutants and in *mir-61; mir-247* double mutants (Table S3), although we did not test the effects of combining these mutants with mutants of *mir-44* and *mir-45*, which have the same seed and thus are predicted to target the same messages. Similarly, *let-60 RAS* has been suggested to be a target of miR-84, based on the observation that overexpression of miR-84 from a transgene suppresses the multivulva phenotype of *let-60 RAS* activation mutants. If *let-60 RAS* is a target of miR-84, loss of *mir-84* might result in *let-60 RAS* overexpression and possibly a multivulva phenotype [91,92]. However, as we reported previously, *mir-84* single mutants or *mir-48 mir-241; mir-84* triple mutants do not have a multivulva phenotype [52]. Thus, for both miR-84 and miR-61, we were unable to confirm a role in vulval development based on loss-of-function alleles. We conclude that these miRNAs are not required for vulval development and suggest that either they act redundantly with other miRNAs or other pathways in vulval development or they do not normally act in vulval development at all.

### Redundancy of miRNAs and Their Regulatory Pathways

One difference between most protein-coding genes and most miRNA genes in C. elegans is the number of paralogs. Whereas fewer than 25% of protein-coding genes have a recognizable paralog in the C. elegans genome [93], about 60% of miRNAs are members of a family of two to eight genes [19]. A higher number of paralogs might be a consequence of smaller gene size, which could allow a greater opportunity for gene duplication. As a consequence, miRNAs might act redundantly with other miRNAs and mutation of all paralogs of a miRNA or a miRNA family might result in synthetic abnormal synthetic phenotypes. Alternatively, some nematode miRNAs might act in parallel with other regulatory pathways that can compensate gene expression when the miRNAs are lost. For example, genetic data indicate that *Drosophila mir-7* directly regulates the transcriptional repressor Yan in the fly eye, but that loss of *mir-7* does not appreciably alter eye development, probably because of redundant protein turnover mechanisms that can also downregulate Yan [58]. In such a scenario, disruptions in the other mechanisms would be needed to reveal the miRNA function.

### Roles for Evolutionary Conserved miRNAs

The discovery that the *let-7* miRNA is conserved among bilateria, including such disparate organisms as C. elegans and humans [94], appears not to have been an exception: for 15 miRNA families, miRNAs with identical seeds have been found in C. elegans, flies, fish, and mammals, and several additional families are predicted to be conserved throughout these diverse lineages [19,95–97]. The conservation is not only for primary miRNA sequences, but also, at least in some cases, for patterns of expression. For example, the miRNA miR-1 is expressed in muscles of *Drosophila*, the zebrafish, and the mouse [11,56,98]. However, the predicted mRNA targets of miRNAs might not share the same degree of conservation as miRNA expression patterns—the spectrum of predicted mRNA targets varies significantly among metazoans [99]. With several miRNA loss-of-function mutants of *Drosophila* now available, we can begin to compare miRNA functions between C. elegans and *Drosophila*. Among the microRNAs for which mutations exist for flies and worms, Dmir-1 and C. elegans miR-1 are the most similar in sequence [56]. Whereas Dmir-1 loss-of-function mutant fly larvae display muscle degeneration and die [56], we found that C. elegans miR-1 loss-of-function mutant animals are fully viable. Despite these differences, the *mir-1* miRNA family could have a conserved role in muscle homeostasis and function. For example, the severity of the muscle defect of *C. elegans mir-1* mutants might depend on physiological conditions, as is the case for the Dmir-1 mutant phenotypes of *Drosophila* [56].

We expect that as additional miRNA mutants become available for flies and other animals there will be future comparative studies of the biological functions of miRNAs using the collection of C. elegans miRNA mutants we have generated. More generally, we believe that the functions of miRNA genes, like the functions of protein-coding genes, will often prove to be conserved among animals, and that the collection of miRNA mutants we have generated will help define, test, and analyze general biological roles of miRNAs.

## Materials and Methods

### Nematode methods.

C. elegans was grown using standard conditions [100]. The wild-type strain was var. Bristol N2 [101]. Nematodes were grown at 25 °C, except where otherwise indicated. Details about the mutant alleles we generated are shown in Table S1. All strains generated in this study have been submitted to the *Caenorhabditis* Genetics Center. Deletion allele information can be accessed directly from WormBase (http://www.wormbase.org).

### Generation of deletion mutants.

Deletion mutants were isolated from a frozen library of worms mutagenized with ethyl methanesulphonate (EMS), 1,2,3,4-diepoxybutane (DEB), or a combination of UV irradiation and thymidine monophosphate (UV-TMP) [82,83]. In most instances, to enhance the detection of deletions one or two “poison” primers were included in the first round of nested PCR reactions [84]. These poison primers were designed to anneal close to the mature miRNA sequence. In the first round of PCR, the three primers in the reaction (external forward, external reverse, and poison primers) generated both a full-length (from external primers) and a shorter product (from external and poison) from the wild-type allele. The shorter product was amplified more efficiently and thereby out-competed the amplification of full-length product. A deletion allele that removed the miRNA sequence and therefore removed the poison primer-binding site generated a product only from the external primers. In the second round of PCR, two internal primers designed just inside of the external primers amplified the full-length product but not the shorter product from the wild-type allele and a single product from the deletion allele. Mutant strains were outcrossed with the wild-type strain as indicated (Table S1).

### Phenotypic analysis.

The minimum number of individual animals scored in each assay is given as *n* in parentheses below. (1) Locomotion: Number of body bends during a 20-s period were counted 4 min after transferring 1-d-old adult animals to fresh plates containing food (*n* = 10). (2) Pharyngeal pumping: Frequency of grinder displacement was counted for 20 s by eye, but otherwise as described previously [102] (*n* = 5). (3) Defecation: The time between defecation cycles marked by posterior body muscle contraction events was measured [103] (*n* = 3, 5 events per animal). (4) Egg laying: 1-d-old adult animals were lysed in bleaching solution for 10 min in the well of a round-bottom 96-well plate, and eggs were counted [100] (*n* = 20). (5) Chemosensory neurons: L2 or L3 larvae were stained with DiO dye (Invitrogen) and filling of the neurons ASI, ASJ, ASH, ASK, AWS, ADL, PHA, PHB was scored [104] (*n* = 15). (6) Cell number/nuclear morphology: L1 larvae were fixed and stained with 4′,6-diamidino-2-phenylindole, dihydrochloride (DAPI) (Invitrogen) as described previously [105]. Nuclei of the ventral cord and intestine were counted [106] (*n* = 15). (7) Dauer development: To assay dauer larva entry, three L4 animals were incubated at 25 °C until the F2/F3 progeny had been starved for at least five days. Animals were washed from plates using 1% SDS in de-ionized H_2_O for 30 min. Dauer larvae were identified by observing their thrashing and re-plated onto plates containing food to assay dauer exit. Constitutive dauer entry was scored by testing animals from plates with food for the presence of dauer larvae isolated after SDS treatment as described above (*n* = 50).

## Supporting Information

Table S1 Deletion Alleles Described in This Study (61 KB XLS)

Table S2 Rescue of Defecation Defect of *mir-240 mir-786* Mutants (35 KB XLS)

Table S3 Normal Induction of 1° and 2° Fates in the Pn.ps of *mir-61* and *mir-247* Mutants (37 KB XLS)

### Accession Numbers

The miRNA sequences discussed in this paper can be found in the miRNA Registry (http://www.sanger.ac.uk/Software/Rfam/mirna/index.shtml). The C. elegans miRNA genes, their genomic location and deletion allele information can de accessed directly from WormBase (http://www.wormbase.org) [107].

## Abbreviations

miRNA: microRNA
